# Calcium Pathways in Human Neutrophils—The Extended Effects of Thapsigargin and ML-9

**DOI:** 10.3390/cells7110204

**Published:** 2018-11-09

**Authors:** Daniela Ribeiro, Marisa Freitas, Sílvia Rocha, José L. F. C. Lima, Félix Carvalho, Eduarda Fernandes

**Affiliations:** 1 LAQV, REQUIMTE, Laboratory of Applied Chemistry, Department of Chemical Sciences, Faculty of Pharmacy, University of Porto, 4050-313 Porto, Portugal; marisafreitas@ff.up.pt (M.F.); silviadgrocha@hotmail.com (S.R.); limajlfc@ff.up.pt (J.L.F.C.L.); 2 UCIBIO, REQUIMTE, Toxicology Department, Faculty of Pharmacy, University of Porto, 4050-313 Porto, Portugal; felixdc@ff.up.pt

**Keywords:** human neutrophils, calcium, thapsigargin, sarco(endo)plasmic reticulum Ca^2+^-ATPase (SERCA), 1-(5-chloronaphthalene-1-sulfonyl)-1H-hexahydro-1,4-diazepine hydrochloride (ML-9)

## Abstract

In neutrophils, intracellular Ca^2+^ levels are regulated by several transporters and pathways, namely SERCA [sarco(endo)plasmic reticulum Ca^2+^-ATPase], SOCE (store-operated calcium entry), and ROCE (receptor-operated calcium entry). However, the exact mechanisms involved in the communication among these transporters are still unclear. In the present study, thapsigargin, an irreversible inhibitor of SERCA, and ML-9, a broadly used SOCE inhibitor, were applied in human neutrophils to better understand their effects on Ca^2+^ pathways in these important cells of the immune system. The thapsigargin and ML-9 effects in the intracellular free Ca^2+^ flux were evaluated in freshly isolated human neutrophils, using a microplate reader for monitoring fluorimetric kinetic readings. The obtained results corroborate the general thapsigargin-induced intracellular pattern of Ca^2+^ fluctuation, but it was also observed a much more extended effect in time and a clear sustained increase of Ca^2+^ levels due to its influx by SOCE. Moreover, it was obvious that ML-9 enhanced the thapsigargin-induced emptying of the internal stores. Indeed, ML-9 does not have this effect by itself, which indicates that, in neutrophils, thapsigargin does not act only on the influx by SOCE, but also by other Ca^2+^ pathways, that, in the future, should be further explored.

## 1. Introduction

**Figure 1:**
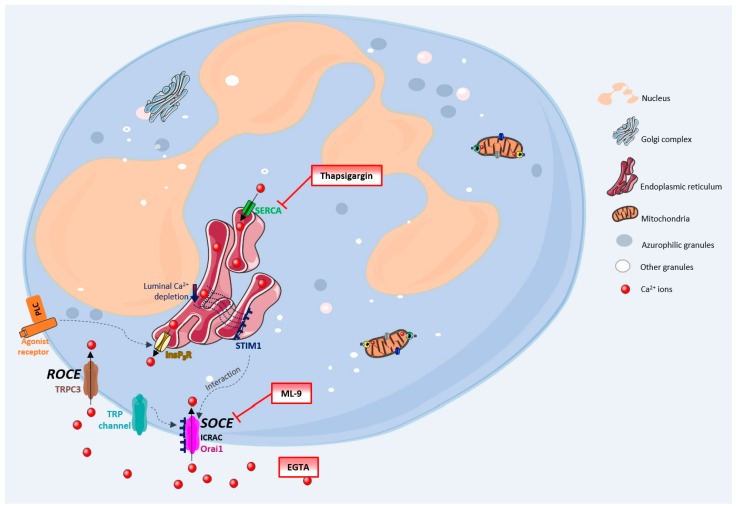
Ca^2+^ pathways in neutrophils. General schematic representation summarizing the mechanisms by which the cytosolic Ca^2+^ concentration is regulated, in human neutrophils, and the mechanisms of action of thapsigargin, ML-9 and EGTA. EGTA-ethylene glycol-bis(2-aminoethylether)-*N*,*N*,*N*’,*N*’-tetraacetic acid; ICRAC-calcium release activated current; InsP3R-inositol-1,4,5-triphosphate receptor; ML-9-1-(5-Chloronaphthalene-1-sulfonyl)-1H-hexahydro-1,4-diazepine hydrochloride; Orai1-Orai protein; PLC-phospholipase C (G-protein linked receptor); SERCA-sarco(endo)plasmic reticulum Ca^2+^-ATPase; STIM-stromal interacting molecule; SOCE-store-operated calcium entry; ROCE-receptor-operated calcium entry; TRP-transient receptor potential; TRPC3-transient receptor potential channel 3.

Calcium is a well-known intracellular second messenger with proven involvement in a wide variety of biological processes, and vital for the correct function of cells, tissues and organisms [1,2]. The Ca^2+^ role in immune cells, especially neutrophils, is of great scientific and therapeutic interest, as it has already been proven that this cation is involved in processes like neutrophils activation, oxidative stress, cell death/clearance and inflammation [3,4,5]. The mechanisms by which the cytosolic Ca^2+^ concentration is regulated have been comprehensively discussed and studied along the years and adjustments of increasing complexity have been made, especially concerning neutrophils. Figure 1 generally summarizes these processes. The Ca^2+^ movements into the cell occur through two closely related events: first, a rapid emptying of the Ca^2+^ stores occur and then some intermediate mechanism translates this information to plasma membrane channels that enable Ca^2+^ entry to refill the depleted stores. This process is generally known as SOCE (store-operated calcium entry) [4,6,7]. In SOCE, the Ca^2+^ influx occurs by the formation of a ternary complex between STIM (stromal interacting molecules), Orai proteins and TRP (transient receptor potential) channels. STIM (STIM1 and STIM2) are Ca^2+^ sensors linking the Ca^2+^ stores to plasma membrane channels. Orai proteins (Orai-1, -2 and -3) are four transmembrane spanning proteins and are electrophysiologically recognized as participating in ICRAC (calcium release activated current) [2,4,5,8,9]. There are various types of TRP channels. In granulocytes it was found the expression of TRPC1, 2, 3, 4, 5 and 6, TRPM2, and TRPV2. However, the link between TRP channels and SOCE stays unclear [10,11]. TRPC1, 3, 4 and 6 proteins are normally expressed in neutrophils’ cell membrane and it is believed that there is a strong association between TRPC3 and SOCE in these cells [5,12].

Despite this broadly accepted mechanism, there may also exist another influx mechanism which is thought to be relatively store independent and receptor dependent, designated as ROCE (receptor-operated calcium entry) [5]. However, this mechanism remains unexplored in human neutrophils. The intracellular signalling molecules required to induce this mechanism are also not currently known [4,9,13]. TRPM2, which is regulated by intracellular adenosine diphosphate ribose (ADPR), and well characterized in neutrophils, is also an important candidate to the ROCE regulation [14].

Once inside the cells, Ca^2+^ can interact with its binding proteins or be sequestered in its stores. These stores are endoplasmic reticulum (ER) or mitochondria. The first one is the largest Ca^2+^ store, with concentrations reaching milimolar levels. Ca^2+^ levels within this store are regulated by: SERCA (sarco(endo)plasmic reticulum Ca^2+^-ATPase) pumps, InsP3 (inositol-1,4,5-triphosphate) receptors, RYRs (ryanodine receptors) and Ca^2+^ binding proteins (calreticulin and calsequestrin). Mitochondria uptakes Ca^2+^ through an electrophoretic uniport transporter, which has low Ca^2+^ affinity, and, as so, its pool under physiological conditions is low. However, this pool considerably increases under pathological conditions. Ca^2+^ flux from the mitochondria to the cytoplasm may occur by: the opening of PTP (permeability transition pore), Na^+^/H^+^-dependent Ca^2+^ exchange or uniporter reversal. The intracellular levels of Ca^2+^ in unstimulated cells are around 100 nM and it is maintained through ER uptake of Ca^2+^ and the efflux to the extracellular space through PMCA (plasma-membrane Ca^2+^-ATPase) or NCX (Na^+^/Ca^2+^ exchanger) [15]. Emerging evidence indicates that Ca^2+^is also stored in other organelles designated as acidic stores due to their acidic interior and the presence of Ca^2+^. These stores include endosomes, secretory granules, acidocalcisomes, vacuoles, lysosomes, lysosome-related organelles, and the Golgi complex [16].

The SOCE mechanism has been studied using SERCA inhibitors (which block the reuptake of Ca^2+^ to the ER), providing a model of store depletion and Ca^2+^ influx activation, but without the participation of receptors and associated biochemical signals [13]. These inhibitors reveal the ongoing Ca^2+^ “leak” from the ER that leads to luminal Ca^2+^ depletion, but without the concomitant generation of InsP3 signals [2]. Thapsigargin, a plant-derived sesquiterpene lactone, is a highly specific and essentially irreversible SERCA inhibitor, and also identified as a tumour promoter [17,18]. It has long been used in the study of intracellular Ca^2+^ flux [19]. Despite the proven utility and extensive use of thapsigargin, its use in neutrophils’ studies is scarce. Neutrophils are essential first response cells of the immune system and thapsigargin could be very useful to better understand Ca^2+^ pathways in these cells. In this sense, this work has its focus on the thapsigargin effects on human neutrophils’ Ca^2+^ routes, adding valuable knowledge to the already known processes.

## 2. Materials and Methods

### 2.1. Reagents

The following reagents were purchased from Sigma-Aldrich Co. LLC (St. Louis, MO, USA): dimethylsulfoxide (DMSO), RPMI 1640 medium, fetal bovine serum, l-glutamine, penicillin, streptomycin, Dulbecco’s phosphate buffer saline, without calcium chloride and magnesium chloride (PBS), trypan blue solution, thapsigargin, 1-(5-chloronaphthalene-1-sulfonyl)-1H-hexahydro-1,4-diazepine hydrochloride (ML-9), ethylene glycol-bis(2-aminoethylether)-*N*,*N*,*N*’,*N*’-tetraacetic acid (EGTA). Vacuum tubes with K_3_EDTA were purchased from Vacutainer Systems (Franklin Lake, NJ, USA). FLUO-4 AM was purchased Life Technologies (Carlsbad, CA, USA). MitoScreen Flow Cytometry Mitochondrial Membrane Potential Detection Kit were purchased from BD Biosciences (San Diego, CA, USA).

### 2.2. Equipment

The performed fluorimetric kinetic analyses were executed in a microplate reader (Synergy HT, BIO-TEK, Winoosky, VT, USA). Each study corresponds at least to three individual experiments, performed in duplicate in each experiment.

### 2.3. Human Neutrophils Isolation

After informed consent of healthy blood donors, in accordance with Helsinki Declaration, venous blood was collected by antecubital venipuncture into K_3_EDTA vacuum tubes, in Centro Hospitalar do Porto-Hospital de Santo António blood bank. Human neutrophils were isolated from blood, according to a previously described density gradient centrifugation method [20]. Neutrophils were resuspended in RPMI 1640 incubation medium [(pH 7.4) supplemented with 10% fetal bovine serum, 2 mM l-glutamine, 100 U/mL penicillin and 0.1 mg/mL streptomycin]. Cell viability and cell yield were evaluated by the trypan blue exclusion method as previously reported [20].

### 2.4. Measurement of Intracellular Free Ca^2+^ Flux

Isolated neutrophils (4 × 10^6^ cells/mL), in RPMI medium, were preincubated with FLUO-4/AM (3 µM), during 30 min, in a humidified incubator, at 37 °C. Cells were then centrifuged at 870× *g*, for 5 min, at 20 °C. The pellet was resuspended in PBS and the cell number readjusted. The monitoring of the thapsigargin effects in the intracellular free Ca^2+^ flux was performed in a microplate reader, by monitoring fluorimetric kinetic readings for 1 h. The reaction mixtures contained human neutrophils (3 × 10^6^ cells/mL), DMSO/PBS (1/1) and thapsigargin (0–32.5 µM), in a final volume of 200 µL. The measurements were carried out at 37 °C, under continuous soft shaking. The excitation and emission wavelengths used were 485 ± 20 and 590 ± 35 nm, respectively. To further understand thapsigargin effects, the SOCE broadly studied inhibitor, ML-9, was used as follows: the reaction mixtures contained human neutrophils (3 × 10^6^ cells/mL), ML-9 (0–117 µM), DMSO/PBS (1/1) and thapsigargin (16.2 µM), in a final volume of 200 µL. The measurements were carried out as previously described. EGTA, a Ca^2+^ chelator, was also used: human neutrophils (3 × 10^6^ cells/mL) were preincubated with EGTA (800 µM), for 5 min, and then ML-9 (0–117 µM), DMSO/PBS (1/1) and thapsigargin (16.2 µM) were added to the reaction mixture, in a final volume of 210 µL.

### 2.5. Viability Assay

The isolated neutrophils (3 × 10^6^ cells/mL) were incubated with thapsigargin (0–32.5 μM), ML-9 (0–117 µM) and EGTA (0–800 µM) in the same reaction conditions described in the intracellular free Ca^2+^ flux assay. After an incubation period time of 1 h, an aliquot of the reaction mixture was added to an equal volume of trypan blue solution 0.4% in a microtube and gently mixed. After 2 min on ice, the neutrophils number was counted in a Neubauer chamber using an optic microscope with the 40× magnification, and the viability calculated. The solvent used for thapsigargin and ML-9 was also tested.

### 2.6. Statistical Analysis

GraphPad Prism™ (version 6.0; GraphPad Software, San Diego, CA, USA) was used to perform the statistical analysis. Results are expressed as mean ± standard error of the mean (SEM). Statistical comparison between groups was estimated using the one-way analysis of variance (ANOVA), followed by the Bonferroni’s post-hoc test. In all cases, *p*-values lower than 0.05 were considered as statistically significant.

## 3. Results

### 3.1. Intracellular Free Ca^2+^ Flux

To better understand thapsigargin effects in the human neutrophils Ca^2+^ flux, five different experimental conditions were tested: (a) thapsigargin alone was incubated with these cells, for 1 h; (b) thapsigargin and ML-9 were simultaneously added and cells were then incubated for 1 h; (c) thapsigargin was incubated with the cells for 30 min, after which ML-9 was added for more 30 min; (d) ML-9 was incubated with the cells for 30 min, after which thapsigargin was added for more 30 min; (e) these same experiments, from (a) to (d), were performed after a preincubation of the cells with EGTA for 5 min.

### 3.2. Thapsigargin Effects

**Figure 2:**
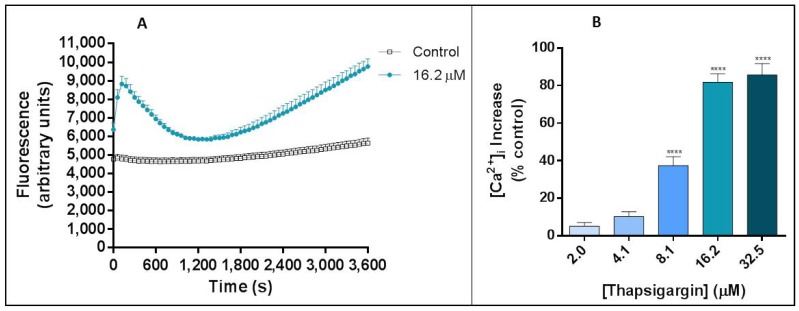
Thapsigargin effects on human neutrophils. (**A**) Thapsigargin (16.2 μM) effects on human neutrophils intracellular free Ca^2+^ concentration, during 1 h. (**B**) Thapsigargin (2.0–32.5 μM)-induced increase of intracellular free Ca^2+^ concentration in human neutrophils, after 1 h of incubation. The values are given as the mean ± SEM (*n* ≥ 3). **** *p* ≤ 0.001, compared with the control assay (without thapsigargin).

Thapsigargin produced a clear fluctuation in the intracellular free Ca^2+^ levels, as expected. These oscillations revealed a very particular pattern (Figure 2A). At time 0 min it is possible to verify a rapid increase of Ca^2+^ levels for around 120 s (2 min), after which it begins a decrease until about 1800 s (30 min). After this, Ca^2+^ levels start to increase again to levels similar to the ones from the first peak. Figure 2B displays the percentage of human neutrophils intracellular Ca^2+^ concentration, when compared with control (without thapsigargin) after 3600 s (1 h) of incubation with thapsigargin. The observed percentage of increase is concentration dependent and statistically significant [*p* ≤ 0.001, compared with the control assay (without thapsigargin and ML-9)], from 8.1 μM (37.4 ± 4.7%) to 32.5 μM (93.8 ± 4.2%).

### 3.3. Thapsigargin and ML-9 Conjugated Effects

**Figure 3:**
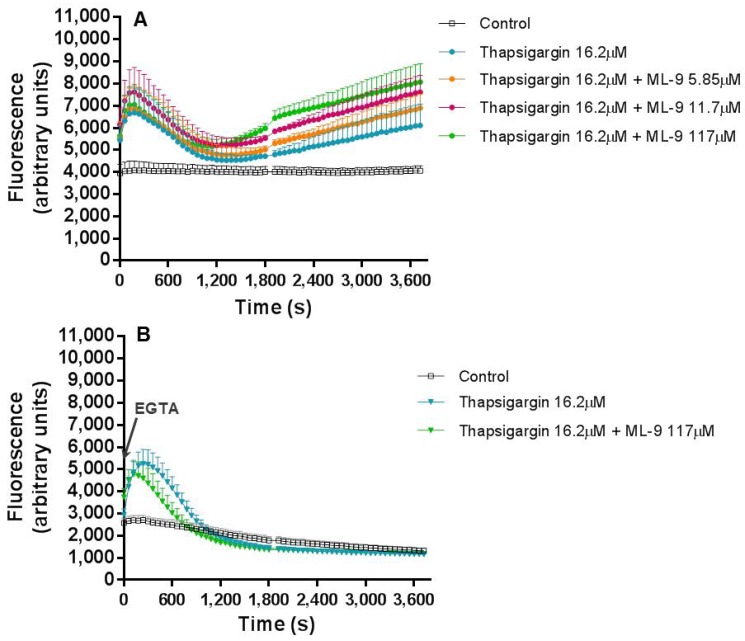
Thapsigargin and ML-9 simultaneous and combined effects. Thapsigargin (16.2 μM) and ML-9 (0–117 µM) combined effects on human neutrophils intracellular concentration of Ca^2+^, during 1 h of incubation time, in the absence (**A**) or presence (**B**) of EGTA (800 µM). The values are given as the mean ± SEM (*n* ≥ 3). The control assay corresponds to the experimental conditions without thapsigargin and ML-9. The figure represents only the most relevant conditions tested.

To better understand the mechanism through which thapsigargin exerts its effects in the Ca^2+^ flux, a broadly described inhibitor of SOCE, ML-9, was used. In a first stage, thapsigargin (16.2 μM) and ML-9 (0–117 µM) were simultaneously added to the cells and their combined effect in the fluctuation of intracellular Ca^2+^ concentration, was monitored during 1 h. The pattern of Ca^2+^ concentration fluctuation along the time was very similar to the one found when thapsigargin was tested alone. However, the intensity of the fluorescence was higher with increasing concentrations of ML-9, indicating an escalation in the intracellular Ca^2+^ concentration (Figure 3A).

When EGTA (800 µM) was added (Figure 3B) to the reactional mixture before any other reagent, in order to chelate the extracellular Ca^2+^, it is obvious the maintenance of the first peak of fluorescence (maintenance of the intracellular Ca^2+^ levels) with the complete disappearance of the second fluorescence escalation, in all the tested conditions, suggesting a clear decrease in the intracellular Ca^2+^ concentration. Interestingly, and contrary to what happened in the absence of EGTA, ML-9 did not induce the increase of the fluorescence intensity of the first peak when compared to thapsigargin alone (Figure 3B).

**Figure 4:**
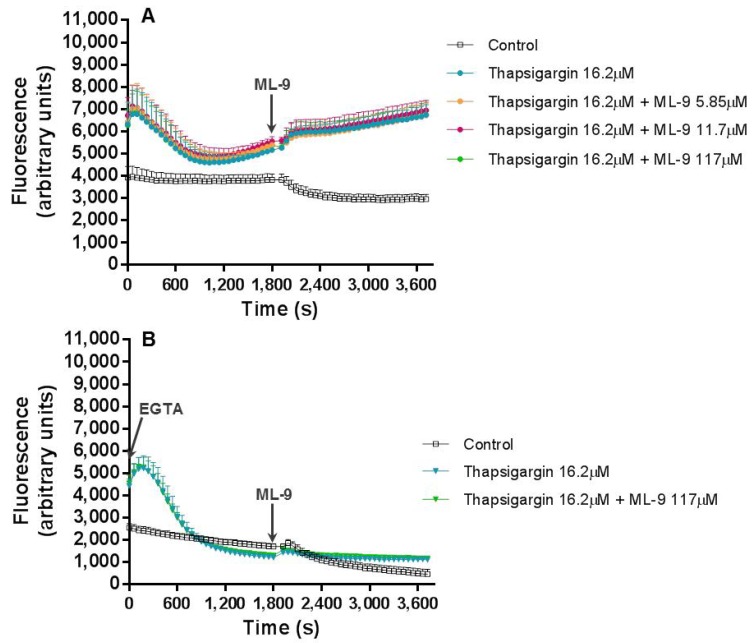
Thapsigargin and ML-9 combined effects. Thapsigargin (16.2 μM) and ML-9 (0–117 µM), added 30 min after the beginning of the experiment, combined effects on human neutrophils intracellular Ca^2+^ concentration, during 1 h of incubation time, in the absence (**A**) or presence (**B**) of EGTA (800 µM). The values are given as the mean ± SEM (*n* ≥ 3). The ‘control’ assay corresponds to the experimental conditions without thapsigargin and ML-9. The figure represents only the most relevant conditions tested.

In a third experiment, we first inhibited SERCA with thapsigargin (16.2 μM) for 30 min. After this time, SOCE was inhibited by ML-9 (0–117 µM). Contrary to the results obtained when thapsigargin and ML-9 were added simultaneously, in this case, the addition of ML-9 after thapsigargin exerted its effects and did not affect the fluorescence increase produced by thapsigargin (Figure 4A). The addition of EGTA (800 μM) produced the same effects described above, with a clear decrease in the intracellular Ca^2+^ concentration, except for the first 300 s (Figure 4B).

**Figure 5:**
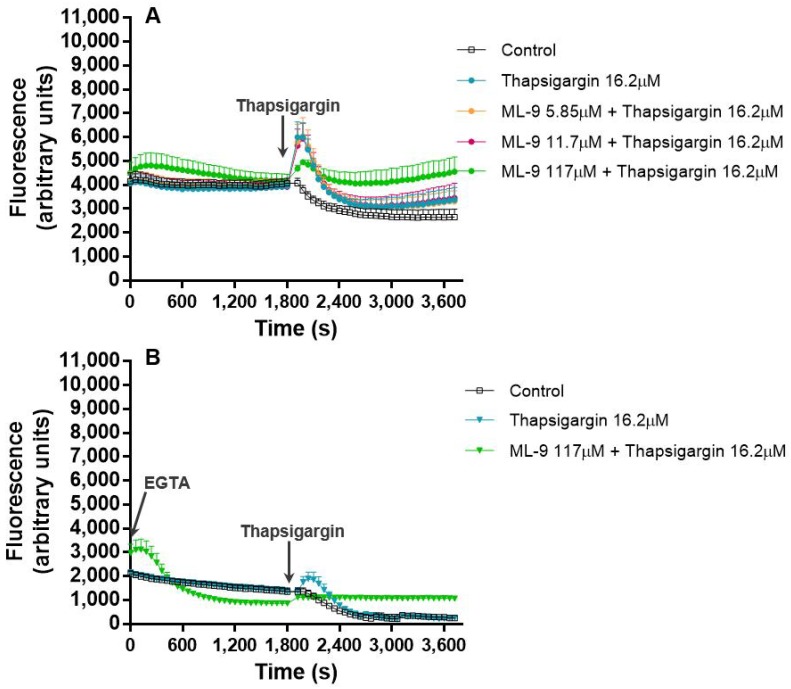
ML-9 and thapsigargin combined effects. ML-9 (0–117 µM) and thapsigargin (16.2 μM), added 30 min after the beginning of the experiment, combined effects on human neutrophils intracellular concentration of Ca^2+^, during 1 h of incubation time, in the absence (**A**) or presence (**B**) of EGTA (800 µM). Values are given as the mean ± SEM (n ≥ 3). The ‘control’ assay corresponds to the experimental conditions without thapsigargin and ML-9. The figure represents only the most relevant conditions tested.

Finally, ML-9 (0–117 µM) was incubated with the cells during 30 min, after which thapsigargin (16.2 μM) was added for 30 min more. Immediately after the addition of ML-9, time 0, a small tendency for an increase of the fluorescence was observed. However, this increment did not achieve statistical significance, either in the presence or absence of EGTA (Figure 5A,B, respectively). In the absence of EGTA, the addition of thapsigargin, 30 min after the beginning of the experiment, clearly led to a statistically significant [*p* ≤ 0.001, compared with the control assay (without thapsigargin and ML-9)] fluorescence increase, in the presence of ML-9 or not (Figure 5A). In the presence of EGTA, a similar peak pattern was only observed for thapsigargin alone, but this increment was not statistically significant (Figure 5B).

### 3.4. Viability

**Figure 6:**
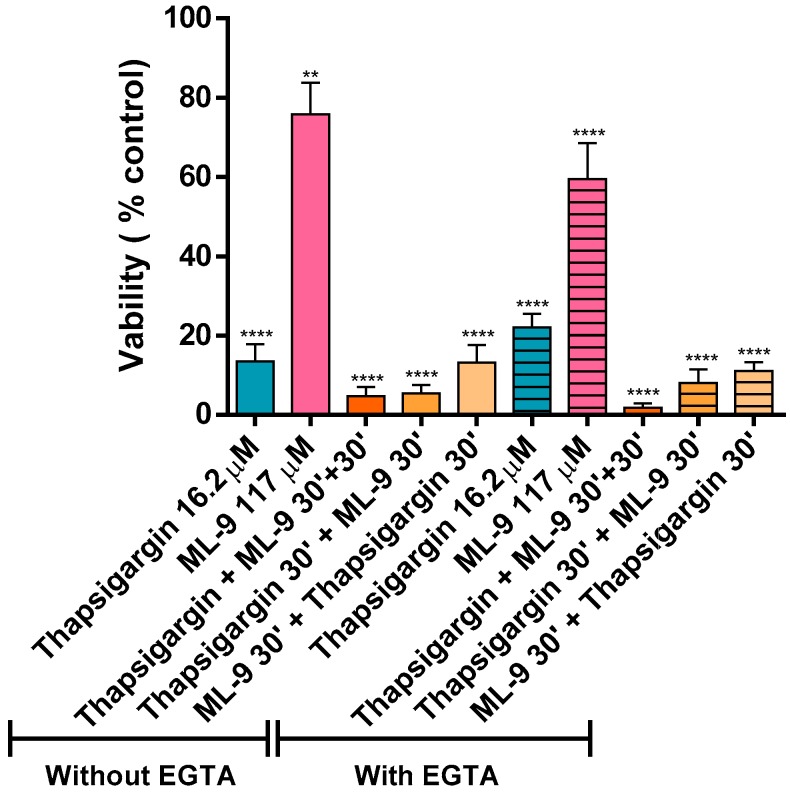
Thapsigargin and ML-9 effects on human neutrophils viability. Effect of 1 h exposure of human neutrophils to thapsigargin (16.2 µM), ML-9 (117 µM) and their mixture on cells’ viability, in the absence or presence of EGTA (800 µM), assessed by the trypan blue exclusion method. Values are given as the mean ± SEM (*n* ≥ 3). ** *p* ≤ 0.01 and **** *p* ≤ 0.001, compared with the control assay (without thapsigargin and ML-9).

Figure 6 displays the effects of thapsigargin and ML-9 (in a total of 1 h incubation) on human neutrophils’ viability. All the tested conditions induced a decrease in neutrophils viability when compared to the control. However, ML-9 induced a lower decrease (75.8 ± 7.8% and 59.5 ± 9.1% viability, without and with EGTA, respectively). Moreover, it is also possible to observe that thapsigargin alone induced slightly less neutrophils’ death than when it was combined with ML-9. When thapsigargin is present from the beginning of the experiment, the viability is lower than when it was introduced 30 min after (e.g., “Thapsigargin + ML-9 30’ + 30’”, 4.7 ± 2.4%, vs. “ML-9 30’ + thapsigargin +30’”, 13.2 ± 4.5%, in the absence of EGTA). Results are not statistically significant when comparing the same conditions with and without EGTA, which means that EGTA does not affect neutrophils viability. The solvent used for thapsigargin and ML-9 was a mixture of DMSO/PBS (1/1) and did not have any effect on the measurements, neither affected the cellular viability (data not shown).

## 4. Discussion

Thapsigargin has long been used to better understand the mechanism of Ca^2+^ pathways. However, in a quick search in PubMed with the keywords “human neutrophils calcium thapsigargin”, only 135 results are obtained and, from these, only approximately 12 are related in some way to the thapsigargin action on neutrophils. It is worth noting that from these 12, only three have their focus on the mechanism of action of thapsigargin in human neutrophils, the works from Salmon et al. [5,9,13].

Therefore, it seemed pertinent to develop a more complete study where the thapsigargin effects were monitored during a longer period of time, as compared to previous studies; without the addition of any external Ca^2+^, to warrant the natural exchanges between cells and the outer medium—resembling more closely the physiological conditions; and with a more sensitive Ca^2+^ probe. The chosen probe, Fluo-4 AM, is an ester form and belongs to the new group of fluorescent indicators with visible excitation and emission wavelengths to measure free cytosolic Ca^2+^. The popularity of Fluo-4 AM has been growing because of its high Ca^2+^ affinity, large dynamic range upon binding, good cell loading properties, and exceptional match of the excitation wavelength with the widely used argon ion laser. Fluo-4 AM dissociation constant for Ca^2+^ is 0.35 μM. This confers Fluo-4 a better resolution for measurements of high Ca^2+^ levels than the one obtained for Fura-2 [21]. Additionally, the present experiments were developed in a microplate reader to enable monitoring Ca^2+^ levels variations throughout time [22].

The effects of thapsigargin alone on the human neutrophils’ Ca^2+^ intracellular levels were monitored for 1 h and a biphasic change in Ca^2+^ levels was observed. This biphasic Ca^2+^ signal generation was already described and it is due to: in a first phase, the export of Ca^2+^ from the ER into the cytosol and in a second phase, the influx of Ca^2+^ from the extracellular medium. This last phase corresponds to the cell response to the depletion of Ca^2+^ stores and to a sustained increase of Ca^2+^ levels [18,23]. In accordance, we observed an immediate increase in intracellular Ca^2+^ levels immediately after the addition of thapsigargin to the cells, and the first peak occurred at 120 s (2 min). After this peak, the Ca^2+^ levels start to decrease until 1800 s (30 min) of exposure. The second pattern of increase starts to appear after this time and it is a sustained signal, even after 60 min. Previous studies also showed a transient intracellular Ca^2+^ concentration increase to a peak value attained in 1 to 2 min that then decline to a lower level [24,25]. Dupont et al. [26], however, concluded that Ca^2+^ entry through SOCE occur quickly, following observation that, after 20 s of thapsigargin addition the Ca^2+^ levels have already reached a third of the maximum value, approximately.

A strategy to better understand the mechanism of action of thapsigargin may be achieved by the addition of other specific inhibitors, especially the ones that act on different pathways. There are various SOCE inhibitors described in the literature. If in one hand, none of these agents is a selective inhibitor, on the other hand they serve as useful probes for testing the involvement of ICRAC in specific responses [27]. In this sense, ML-9 was chosen as a broadly used inhibitor of SOCE. The precise molecular target of this piperazine compound is not yet fully disclosed, but it is clear the involvement of myosin light-chain kinase (MLCK) inhibition [9]. In this sense, we adopted three strategies: (a) thapsigargin and ML-9 were simultaneously added and incubated with the cells, for 1 h; (b) thapsigargin was incubated with the cells in the first 30 min, after which ML-9 was added for more 30 min; (c) ML-9 was incubated with the cells during the first 30 min, after which thapsigargin was added for more 30 min. These same experiments, from (a) to (c), were performed after a preincubation of the cells with EGTA for 5 min, to guarantee the elimination of the contribution of all extracellular Ca^2+^ and to conclude about the origin of the Ca^2+^ that generate the intracellular concentration fluctuations.

Beginning with experiment (a), where thapsigargin and ML-9 were simultaneously added to the cells, and their effect in the Ca^2+^ levels fluctuation during 1 h was registered. By adding these two compounds simultaneously we were theoretically inducing SOCE, by ER store depletion, with thapsigargin, and, at the same time, inhibiting SOCE with ML-9. In reality, their added effects were translated in exactly the same Ca^2+^ biphasic fluctuation pattern of thapsigargin alone. However, it was interesting to observe that higher concentrations of ML-9 induced higher, and statistically significant fluorescent signals [*p* ≤ 0.001, compared with the control assay (without thapsigargin and ML-9)], both at the beginning of the experiment and at the second phase of Ca^2+^ increase. Considering that ML-9 inhibits SOCE, it was expected that the second increase (after the first 30 min) would not appear, because it is believed to be due to the influx of Ca^2+^ from the extracellular medium. However, such effect was not observed. In fact, the characteristic thapsigargin effect is still observed and it seems that ML-9, in some way, has a synergic effect, because ML-9 alone does not alter the Ca^2+^ levels when compared to the control (data not shown). In the presence of EGTA, i.e. without extracellular Ca^2+^, the first peak is preserved, meaning that this Ca^2+^ comes from the intracellular stores. The second peak completely disappears, indicating that the second peak was caused by the entry of Ca^2+^ from the extracellular medium to neutrophils cytoplasm. This behaviour was observed for all the conditions, so even in the presence of ML-9, the origin of the Ca^2+^ was the same, the intracellular stores.

In experiment (b), thapsigargin was incubated with the cells in the first 30 min, after which ML-9 was added for another 30 min. In the first part of the experiment, all occurred as expected. After ML-9 addition, the intracellular Ca^2+^ levels were exactly the same with or without ML-9; contrary to what we registered in experiment (a), where these values were increased with higher ML-9 concentrations. Moreover, in the presence of EGTA, the first fluorescence peak was maintained, as expected, and the second increase disappeared, meaning once more the participation of extracellular Ca^2+^ in this last effect. This leaves a doubt, ML-9 which inhibits Ca^2+^ entry into cells by SOCE, does not alter the signal, but EGTA does. We may argument that ML-9 concentrations were not high enough and in this sense did not inhibit SOCE, as expected. However, ML-9 has been used in some other similar works in concentrations of 50 and 100 µM [13]; the maximum concentration used in this work was 117 µM. Still, if there was no extracellular Ca^2+^, thapsigargin is only able to elevate Ca^2+^ levels via intracellular stores, but not via an extracellular Ca^2+^ influx. As such, we may state that once thapsigargin had activated SOCE, ML-9 is not capable to reverse this effect or that thapsigargin induce Ca^2+^ entry through a different pathway from SOCE, for example ROCE. Indeed, it is believed that ML-9 inhibits thapsigargin-induced SOCE but not ROCE [9]. Interestingly, Pantaler et al. [14] developed a work with human neutrophils and concluded that thapsigargin should not be considered a specific and exclusive tool to SOCE activation, when other Ca^2+^-activated pathways such as TRPM2 are present, which is the case in human neutrophils, corroborating the results obtained in the present work.

In the last experiment, (c), ML-9 was incubated with the cells during the first 30 min, after which thapsigargin was added for more 30 min. In the first 30 min, immediately after its addition, ML-9 seemed to incite an increase in the Ca^2+^ levels, but it was not statistically significant. However, it means that ML-9 may induce the increase of intracellular Ca^2+^ levels. Indeed, in the presence of EGTA, this peak remains, meaning that this increase is due to the Ca^2+^ release from the internal stores. From this, two hypotheses may be drawn: ML-9 may exert its action on SOCE really quickly and neutrophils try to compensate it, as also indicated in experiment (a), or ML-9 may act on another level of the Ca^2+^ pathways and not only via SOCE. In the absence or presence of EGTA, when thapsigargin is added to the reactional mixture, without ML-9, an immediate increase of the fluorescent signal is seen. This means the first increase in the Ca^2+^ levels incited by thapsigargin is still and totally due to the release of Ca^2+^ from the internal stores (EGTA does not affect this increase). Immediately after this peak the signal intensity starts to decline and then we can see a tendency to start improving again, which corresponds to the typical effect of thapsigargin. In the presence of ML-9 it is also obvious that after the addition of thapsigargin a first peak appears, and it is similar to all conditions, but, once more, there is a tendency to a signal increase with increasing concentrations of ML-9. It is noteworthy that this last effect is completely relapsed when EGTA is present, proving the involvement of extracellular Ca^2+^ in this signal; it is possible that Ca^2+^ is entering through another path. In 2011, Salmon et al. [9] reviewed the pharmacology of ROCE in human neutrophils. These authors stated that nowadays it is believed that ML-9 directly interferes with SOCE elements, namely STIM1. Moreover, they reported that human neutrophils preincubation with ML-9 (before thapsigargin) induce a marked increase in Ca^2+^ release and influx. This effect may be due to the ML-9-induced opening of the InsP3 Ca^2+^ release channels; concluding that ML-9 inhibits SOCE but not ROCE [9]. This conclusion may contribute to explain the results obtained in our experiments.

Salmon et al. developed a study in human neutrophils [13] to evaluate the thapsigargin mechanism of action, using Fura-2 AM as probe and ML-9, accompanying their effects only during 540 s (9 min) [13]; however, they always added EGTA (1 mM) to the neutrophils suspension at the start of the experiments because they only wanted to evaluate the contribution of the Ca^2+^ from the internal stores. Afterwards (341 s) they added CaCl_2_ (2 mM) to the medium. The authors also performed the experiment, adding thapsigargin first, and then ML-9 and the other way around, but they did not add the two compounds at the same time as we did in the current work. So, with all extracellular Ca^2+^ chelated, they added thapsigargin (1 µM) to the neutrophils and they observed a signal increase, reaching its maximum at 341 s, as we did, but the peak that we observed was at 120 s. This discrepancy may be explained by the divergent experimental conditions. When ML-9 (100 µM) was added, at 341 s, a slight increase of the signal was registered. Then the authors added CaCl_2_ to the medium and an increase on the signal, or intracellular Ca^2+^ concentration, was observed, but in a lower extent when ML-9 was present. In this work we did not add CaCl_2_ to the reactional mixture, but instead, we worked with the Ca^2+^ concentration already existent and this effect of ML-9 was not seen, even in the same period of time. Further ahead they did another experiment, in which ML-9 was added to the neutrophils, with all extracellular Ca^2+^ chelated, and observed a signal increase, and this signal was higher when thapsigargin was added-ML-9 enhanced thapsigargin-induced Ca^2+^ stores emptying [13]. This addition of effects, corroborate our results; however, in the present work, this response was observed much later in time and was more evident in the second phase of Ca^2+^ levels increase or when the two compounds were added at the same time. The authors [13] also refer that ML-9 is described as an inhibitor of thapsigargin-induced Ca^2+^ release and the following Ca^2+^ influx in various culture cell lines. In the present work, in human neutrophils, this last ability of ML-9 was not corroborated in any of the tested conditions. In fact, it is known that ML-9 inhibits SOCE through the interference with STIM1 localization, but its specific target and mechanism of action are unknown [2]. Moreover, it has been reported that ML-9 increases cytoplasmic Ca^2+^ through its release from InsP3-sensitive stores [9]. This explanation may be valid to the results obtained when ML-9 was in contact with the neutrophils from the beginning of the experiments [(a) and (c)].

The cell viability was evaluated after an hour of incubation in the already described conditions. All the tested conditions led to a statistically significant loss of viability [** *p* ≤ 0.01 and **** *p* ≤ 0.001, compared with the control assay (without thapsigargin and ML-9)]. As above mentioned, Ca^2+^ has an extremely relevant role in cellular processes, namely in cell death. In recent years it has become obvious that Ca^2+^ can activate distinct paths of cell death mechanisms. There are several theories [15] about the relation between cell death and variations of Ca^2+^ fluxes. Even that induction of apoptosis also occurs in the absence of Ca^2+^ fluxes. It is commonly accepted that severe dysregulation of Ca^2+^ fluxes lead to necrosis and that more controlled increases lead to apoptosis. So, subtler changes of Ca^2+^ may have different effects on cell death modulation, as in cell proliferation and differentiation, for example. It is now known that Ca^2+^ related processes are intimately connected to caspases, the apoptosis effectors. Under stress response, ER can trigger apoptosis. The ER stress may be triggered by changes in the protein folding due to Ca^2+^ overload or depletion of the Ca^2+^ pool. ATP levels and mitochondrial function are also vital to influence the death mode [1,15,23]. Furthermore, Ca^2+^-related processes can also trigger the elimination of death cells by phagocytosis or by their lysis. This process of cell death is even more important when we talk about neutrophils [15]. Indeed, the clearance of these cells is crucial to avoid the further propagation of an inflammatory process, for example [18]. Curiously, an antiapoptotic effect of Ca^2+^ is also described, but it is not conclusively identified [15].

It was obvious that thapsigargin led to a more drastic loss of cell viability even when ML-9 was present. However, it was clear that during the first 30 min of incubation with just ML-9, and only after the addition of thapsigargin, this loss was lower. The thapsigargin-induced neutrophils death is known [28]. High concentrations of thapsigargin promote mitochondrial permeability transition (MPT) precisely because the increase of cytoplasmic Ca^2+^ level leads to the accumulation of mitochondrial Ca^2+^. Another hypothesis is that thapsigargin inhibits mitochondrial Ca^2+^ influx, via Ca^2+^ uniporter, and in this way Ca^2+^ efflux is not unimpeded. Indeed, these two mechanisms: cytosolic Ca^2+^ increase and MPT induction may be responsible for the known thapsigargin-induced cell death [29]. It is also described that thapsigargin induces a significant membrane depolarization in polymorphonuclear leukocytes. Indeed, this depolarization is observed after 15 min of exposure and remain even after 30 min [18,23]. Noteworthy, loss of cellular viability was not significant when comparing the experiments in the presence or absence of EGTA. All these results rule out the contribution of extracellular Ca^2+^. Finally, one should keep in mind the intrinsic correlation between Ca^2+^ variations and cell viability [30,31], which may also contribute, at least partially, to the sustained long term observations in the present study.

In conclusion, the obtained results corroborate the general thapsigargin-induced intracellular pattern of Ca^2+^ fluctuation, but it was also observed a much more extended effect in time and a clear sustained increase of Ca^2+^ levels due to the influx by SOCE. Moreover, it was obvious that ML-9 enhanced the emptying of the internal stores. Indeed, ML-9 does not have this effect by itself, which indicates that, in human neutrophils, thapsigargin does not act only on the influx by SOCE, but also by other Ca^2+^ pathways; this should be further explored in the future.
